# Clinical guidelines for early hepatocellular carcinoma treatment options: a systematic review and bibliometric analysis

**DOI:** 10.1097/JS9.0000000000001950

**Published:** 2024-07-23

**Authors:** Chun-Ying Wu, Lee-Yuan Lin, Teng-Yu Lee, Yao-Chun Hsu, Chun-Chieh Yeh, Chiehfeng Chen, Yi-No Kang, Tsai-Wei Huang

**Affiliations:** a Institute of Biomedical Informatics, National Yang Ming Chiao Tung University; b Health Innovation Center, National Yang Ming Chiao Tung University; c Microbiota Research Center, National Yang Ming Chiao Tung University; d Division of Translational Research, Taipei Veterans General Hospital; e Department of Public Health, China Medical University, Taichung; f School of Medicine, College of Medicine, Taipei Medical University; g School of Respiratory Therapy, College of Medicine, Taipei Medical University, Taipei; h Division of Gastroenterology and Hepatology, Taichung Veterans General Hospital; i Department of Medicine, Chung Shan Medical University, Taichung; j Division of Gastroenterology and Hepatology, E-Da Hospital/I-Shou University, Kaohsiung; k School of Medicine, China Medical University Hospital; l Department of Surgery, China Medical University Hospital, China Medical University; m Department of Surgery, Asian University Hospital, Asian University, Taichung; n Department of Public Health, College of Medicine, Taipei Medical University, Taipei; o Division of Plastic Surgery, Department of Surgery, Wan Fang Hospital; p Cochrane Taiwan, Taipei Medical University, Taipei; q Evidence-Based Medicine Center, Wan Fang Hospital, Taipei Medical University; r Research Center in Nursing Clinical Practice, Wan Fang Hospital, Taipei Medical University; s Department of Nursing, Wan Fang Hospital, Taipei Medical University; t School of Nursing, College of Nursing, Taipei Medical University, Taipei, Taiwan

**Keywords:** guideline, hepatocellular carcinoma, liver cancer, radiofrequency ablation, recommendation, RFA, surgical resection

## Abstract

**Background:**

Hepatocellular carcinoma remains a major cause of cancer-related mortality worldwide, with treatment options including radiofrequency ablation (RFA) and surgical resection. This study evaluates the evolving guidelines for these treatments to identify the current consensus and divergences.

**Method:**

The authors conducted a systematic review following PRISMA 2020 guidelines of documents from 2017 to 2024 by major liver societies. The AGREE-II framework assessed guideline quality. This study is registered with PROSPERO (CRD42022342266).

**Results:**

The authors analyzed 23 guidelines and noted significant shifts in treatment recommendations over recent updates. This analysis reveals an increasing endorsement of RFA for certain patient groups and sustained strong support for surgical resection based on robust evidence levels. All demonstrated high quality, with the 2023 Japan Guidelines receiving the highest AGREE-II score. A significant finding was the low level of stakeholder involvement in the development of guidelines.

**Conclusion:**

The study highlights the dynamic nature of clinical guidelines for early-stage hepatocellular carcinoma, underscoring the need for ongoing updates and direct, high-quality comparative studies. The evolving recommendations for RFA, especially its role in managing small, localized tumors, reflect its emerging importance in the treatment paradigm.

## Graphical abstract

**Figure:**
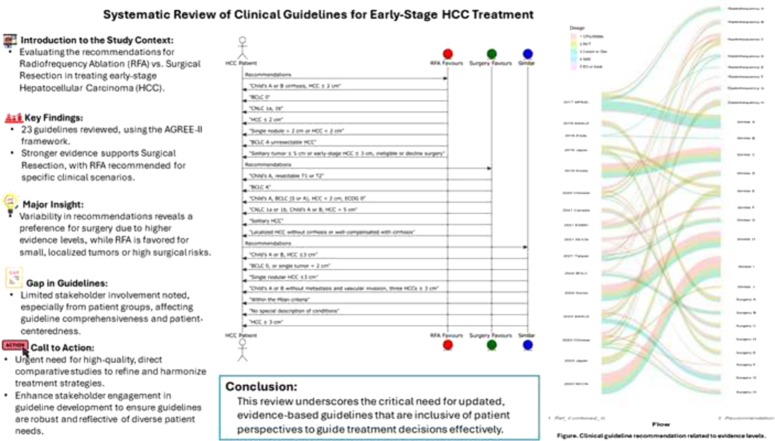

## Introduction

Highlights

- Analyzed 23 guidelines for early hepatocellular carcinoma, focusing on radiofrequency ablation and surgical resection, using AGREE-II framework.
- Assessed guideline quality using the AGREE-II framework, revealing high overall quality but noting deficiencies in stakeholder engagement.
- Identified 26 distinct recommendations demonstrating a preference for surgical resection supported by stronger evidence, while also acknowledging the growing role of radiofrequency ablation for specific clinical scenarios.
- Uncovered variability in guideline recommendations, highlighting the need for direct, high-quality comparative studies to clarify treatment choices.
- Emphasized the importance of inclusive stakeholder involvement in the development of guidelines to enhance their relevance and patient-centeredness.

Hepatocellular carcinoma (HCC) remains a leading cause of cancer-related deaths worldwide, influenced by diverse etiological factors that vary significantly across different regions^1^. The overall 5-year survival rate for liver cancer is about 18%, making it one of the deadliest cancers, second only to pancreatic cancer^2^. Global incidence is on the rise, with projections estimating nearly one million new cases annually by 2025^3^. The etiology of HCC is complex and region-specific; in areas like sub-Saharan Africa and East Asia, hepatitis B virus (HBV) is a predominant risk factor, whereas in Western countries, factors such as hepatitis C virus (HCV), alcohol consumption, and obesity are more prevalent^4–6^. Additionally, the rising prevalence of nonalcoholic steatohepatitis (NASH) highlights the evolving nature of risk factors, particularly in regions with increasing rates of obesity^7^. Early detection of HCC can significantly improve prognosis, with surgical resection offering 5-year survival rates above 70%. However, the majority of patients are diagnosed at an advanced stage, underscoring the importance of prioritizing early detection and effective management to enhance treatment outcomes^8^.

In response to the need for guidance in the treatment and management of early-stage HCC, several leading organizations have issued guidelines. These include the American Association for the Study of Liver Diseases (AASLD)^9^, the European Association for the Study of the Liver (EASL)^10^, the Asian Pacific Association for the Study of the Liver (APASL)^11^, and the National Comprehensive Cancer Network (NCCN)^12^. Encompassing a wide array of topics, these guidelines address early detection, diagnostic methods, staging, and various treatment modalities such as surgical resection, liver transplantation, and various local ablative therapies such as percutaneous radiofrequency ablation (RFA) and microwave ablation. Additionally, they cover transarterial therapies such as transarterial chemoembolization and radioembolization. Adhering to these guidelines empowers healthcare providers to make well-informed decisions, ensuring the delivery of the highest standard of care for patients diagnosed as having early-stage HCC.

Among the treatment modalities for early HCC, RFA, and surgical resection are critical^13^. RFA, a minimally invasive technique, utilizes thermal energy to obliterate cancer cells. A probe, guided by imaging, is inserted into the tumor to emit radio waves that generate heat and induce coagulative necrosis, effectively destroying the tumor^14^. Recommended for patients with small tumors in areas unsuitable for resection, RFA offers a viable alternative^15^. Surgical resection, conversely, entails removing the tumor and a margin of healthy tissue, often considered curative in early-stage HCC when there is no metastasis^16^. The extent of resection is determined by factors such as the size and location of the tumor and the residual liver function of the patient^17^. The decision between RFA and surgical resection for HCC is guided by factors such as tumor size, number, location, overall liver function, and patient health^20^. In some cases, a strategic combination of treatments—such as RFA followed by surgical resection or liver transplantation—may be considered to enhance patient outcomes, leveraging the strengths of each approach^18,19^.

Despite the plethora of guidelines, consensus documents, meta-analyses, and randomized controlled trials (RCTs) on managing early-stage HCC, inconsistencies in recommendations due to varying evidence assessments or scopes present a practical challenge for clinicians. This study aims to systematically review and evaluate the methodological quality and evidence strength of clinical guidelines for early-stage HCC treatment, with a focus on RFA and surgical resection to identify consistencies and divergences, with a novel approach of visualizing our findings to enhance understanding and applicability. It is crucial to emphasize that our objective is not to advocate for the superiority of one treatment modality over another. Instead, we aim to illuminate the varying degrees of consensus and divergence within existing guidelines, revealing areas where further clarity and agreement are needed.

## Methods

### Eligibility criteria

The systematic review specifically focused on guidelines from European and Asia-Pacific hepatology societies due to their significant impact on global clinical practices in the treatment of HCC. These regions were selected because they host key hepatology societies such as EASL and APASL, whose guidelines influence a wide range of associated practices worldwide. Additionally, we included guidelines from the American Association for the Study of Liver Diseases (AASLD) to encompass influential perspectives that shape global standards. The inclusion criterion was a classification of stage 0 and A according to the Barcelona Clinic Liver Cancer (BCLC) staging system. These early-stage cases were characterized by the presence of up to three tumors, each measuring less than 3 cm, and patients with substantial liver function (Child-Pugh A or B). Moreover, the eligible guidelines encompassed cases without macrovascular invasion, extrahepatic spread, or cancer-related symptoms (performance status 0)^23^. Guidelines that did not address the comparison between RFA and surgical resection were excluded from the qualitative syntheses. Only guidelines written in English were included. Disagreements were resolved through adjudication by an independent reviewer. This study protocol was registered with PROSPERO, which is an international online prospective registry of systematic reviews (CRD42022342266).

### Guideline and consensus selection

We selected guidelines published between 2017 and 2024 for inclusion in this study according to the PRISMA guidelines (Supplemental Digital Content 1, http://links.lww.com/JS9/D131, Supplemental Digital Content 2, http://links.lww.com/JS9/D132)^21^ and assessing the methodological quality of systematic reviews (AMSTAR) (Supplemental Digital Content 3, http://links.lww.com/JS9/D133) Guidelines^22^. However, since many HCC guidelines were updated between 2023 and 2024, our review will add revised guidelines during the revision period to make the entire article more complete. To ensure a comprehensive and systematic approach to gathering data, we conducted a detailed search across multiple databases, including PubMed, Embase, Scopus, and the Cochrane Library, covering publications from January 2017 to March 2024. Specific search terms used, included hepatocellular carcinoma, HCC, liver, cancer, treat, manage, guideline, consensus, and recommend, combined with Boolean operators for thoroughness. The documentation of sources was meticulously managed using EndNote and Excel, where each guideline’s full citation, abstract, and relevancy were recorded and reviewed for inclusion based on our criteria. This rigorous process was designed to capture the most relevant and recent information guiding the treatment of HCC. Guidelines or consensus documents focusing on the comparison between RFA and surgical resection for early HCC were included in the analysis. In cases where guideline developers updated their guidelines in a modular manner—where they addressed specific topic areas and published them separately—these updates were considered part of a singular and comprehensive guideline that includes all the updates. The Appraisal of Guidelines for Research and Evaluation (AGREE) evaluated and rated all the related publications as a cohesive entity, acknowledging their interdependence and the unified guidance they provide. Titles and abstracts underwent screening, and full-text papers were retrieved and independently reviewed by two reviewers by using predetermined criteria^23^.

### Information extraction

To analyze early HCC treatment recommendations, a method of textual descriptive synthesis was employed. Two authors independently reviewed the guidelines, extracting relevant information such as the name, publication year, country, organization, status, and funding source of each guideline. Additionally, the level of evidence and grade of recommendation provided in the guidelines were recorded. The specific recommendations for the selection between RFA and surgical resection in patients with early HCC were also extracted, along with the references supporting these recommendations, for evaluating the evidence level. The recommendations were divided into three distinct categories: (a) those advocating surgical intervention, (b) those endorsing radiofrequency-based treatments, and (c) those demonstrating a degree of concurrence in advocating both surgical and radiofrequency procedures.

### AGREE-II evaluation

Seven independent reviewers used the December 2017 version of the AGREE II to assess the quality of each guideline^23^. This instrument serves as a reliable and trustworthy tool for evaluating the rigor, transparency, and clarity of guidelines. It comprises 23 items grouped into six domains, encompassing the guideline’s scope and purpose, stakeholder involvement during development, methodological rigor in evidence gathering and recommendation formulation, linguistic clarity, guideline applicability, and whether issues of potential conflicts of interest are addressed and whether the development group is independent. Each item was scored on a 7-point scale, where 1 indicated strongly disagree and 7 indicated strongly agree. The scores for each item were then averaged among the seven reviewers to determine the overall quality of the guidelines.

In our evaluation of clinical practice guidelines, the assessment team comprised four clinical physicians, two evidence-based medicine experts, and one guideline development specialist. We quantified the inter-rater reliability among these seven evaluators using the Intraclass Correlation Coefficient (ICC). For the average measures, the ICC under the assumption of random raters (ICC2k) was 0.97, demonstrating a high level of consistency among raters, with a CI ranging from 0.95 to 0.99. Similarly, under the assumption of fixed raters (ICC3k), the ICC was 0.98, indicating even greater reliability, with a CI from 0.96 to 0.99. These results confirm a high degree of agreement among evaluators, ensuring that our assessments of guideline quality are robust and reliable.

### Analysis

We systematically analyzed the qualitative information by using tabulation, wherein we extensively compared the characteristics of the guidelines and the criteria or situations associated with each recommendation. Additionally, we employed Sankey diagram analysis to visualize the flow from evidence to recommendations. The graphical approach offers an intuitive sense of the context and quality of each recommendation. To provide a more comprehensive understanding, we also presented the flow from evidence to recommendations based on the present study’s design. The Sankey diagram analysis was performed using the *geom_parallel_sets()* function after data processing with the function *gather_set_data()* in the R package *ggforce*. Furthermore, we used the *χ* ^2^ test for examining the differences in the categories of recommendations based on the level of evidence and study design. The overall *P*-value of the *χ* ^2^ test was computed using a rigorous method through Monte Carlo simulation with 10 000 replicates.

We assessed the strength of evidence supporting clinical guidelines using the Joanna Briggs Institute (JBI) Levels of Evidence^24^. This classification system ranges from Level 1, which includes experimental designs such as RCTs and their systematic reviews, to Level 2, covering quasi-experimental designs like prospective controlled studies. Level 3 encompasses analytical observational studies, including cohort and case–control studies. Level 4 is designated for descriptive observational studies, such as case series and cross-sectional analyses. Finally, Level 5 comprises expert opinions and bench research, including consensus statements and theoretical research. To gain more comprehensive insight into the contextual nuances associated with each recommendation category, we conducted additional proportional tests for each of them. Pairwise comparisons were further performed to detect the presence of any significant difference in the proportion of each JBI Level of Evidence in each recommendation category. For the proportional tests, the *χ* ^2^ test was conducted using the *chisq.test()* function with the parameters *B* and *parameter simulate. P-value* for computing *P*-values through Monte Carlo simulation. For the pairwise test, the function *chisq.theo.multcomp()* was used, with the argument *bonferroni* assigned for the parameter *P.method* in the *RVAideMemoire* package.

## Results

### Characteristics of the included guidelines and consensuses

**Figure 1:**
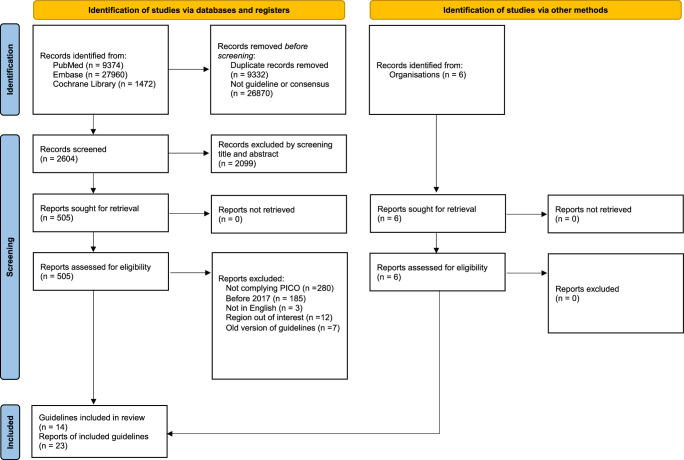
Study selection process.

Figure 1 illustrates the PRISMA flow chart for the article screening and selection processes^21^. Our study included 23 reports from 14 guidelines. Table 1 presents the characteristics of each guideline included in the study. The guideline development groups of the 16 articles originated from various regions, including the United States (8), Europe (4), the Asia-Pacific region (2), Canada (1), China (2), Korea (2), Japan (2), and Taiwan (2)^9–12,18,25–41^. Notably, the AASLD and EMSO guidelines are each presented in two initial publications, for a total of 14 comprehensive guidelines. Although all guideline developers conducted systematic literature searches, the methods for evaluating evidence and grading recommendations varied among them. Most guidelines utilized GRADE (7), BMJ 1999 (1), NCCN categories of evidence and consensus (1), Oxford Centre for Evidence-Based Medicine Levels of Evidence (1), or the Adapted Infectious Diseases Society of America-United States Public Health Service Grading System (1) to grade both study evidence and recommendation strength. However, a few guidelines did not specify the grading system used (3). From 2023 to 2024, AASLD, ASCO, Chinese, Japan, Korea, NCCN, and Taiwan^42^ updated their guidelines. The funding source, or lack thereof, was disclosed in all guidelines except for the APASL guideline. The AGA, APPLE, and ASCO guidelines did not discuss the preference between RFA and surgical resection and were therefore excluded from the qualitative syntheses.

**Table 1:** Characteristics of the included hepatocellular carcinoma management guidelines (*n*=23).

Guideline (years)	Guideline name (s)	Comparing RFA and LR (Yes or No)	Organization, country, or region	Level of evidence included	Grade of recommendation
AASLD (2023, 2018, 2018)	Prevention, diagnosis, and treatment/ Treatment/Diagnosis, Staging, and Management	Y	American Association for the Study of Liver Diseases (AASLD), America	GRADE/level 1-5; high, medium, low, very low	GRADE/strong, weak; strong, conditional
AGA (2022)	Systemic Therapy	N	American Gastroenterological Association (AGA), America	GRADE/high, medium, low, very low	GRADE/strong, conditional
APASAL (2017)	Management	Y	The Asian Pacific Association for the Study of the Liver (APASL), the Asia-Pacific region	GRADE/A,B,C	GRADE/1,2
APPLE (2020)	Treatment of Intermediate-Stage HCC	N	The Asia-Pacific Primary Liver Cancer Expert (APPLE), the Asia-Pacific region	No information	No information
ASCO (2024, 2021)	Systemic Therapy for Advanced HCC	N	American Society of Clinical Oncology (ASCO), America	GRADE/high, medium, low, very low	GRADE/high, moderate, low
BCLC (2022)	Prognosis prediction and treatment	Y	The Barcelona Clinic Liver Cancer (BCLC) group, Europe	No information	No information
Canada (2021)	Hepatocellular Carcinoma	Y	Alberta Health Services, Canada	No information	No information
Chinese (2023, 2020)	Diagnosis and Treatment	Y	The Society of Liver Cancer of China, China	GRADE/high, moderate, low, and very low	GRADE/high, moderate, weak; Oxford Centre for Evidence-Based Medicine Levels of Evidence (2011)
ESAL (2018)	Management	Y	European Association for the Study of the Liver (EASL) and the European Organization for Research and Treatment of Cancer (EORTC), Europe	GRADE/high, medium, low	GRADE/strong, weak
ESMO (2018, 2021)	Management/diagnosis, treatment and follow-up/treatment	Y	ESMO Guidelines Committee, Europe	Adapted Infectious Diseases Society of America-United States Public Health Service Grading System/I, II, III, IV, V	Adapted Infectious Diseases Society of America-United States Public Health Service Grading System/A,B, C, D, E
Japan (2023, 2019)	HCC	Y	The Japan Society of Hepatology (JSH), Japan	GRADE/no mention of the evidence level	GRADE/no mention of the evidence level (2019)
Korea (2022, 2019)	Management	Y	The Korean Liver Cancer Association (KLCA)–NCC Korea Practice Guideline Revision Committee (KPGRC), Korea	GRADE/high (A), medium (B), low(C)	GRADE/strong (1), weak (2)
NCCN (2024, 2021)	Hepatocellular Carcinoma/Hepatobiliary Cancers	Y	The National Comprehensive Cancer Network (NCCN), America	NCCN categories of evidence and consensus/1, 2A, 2B, 3	NCCN categories of evidence and consensus/1, 2A, 2B, 3
Taiwan (2024, 2021)	Management, Surveillance, Diagnosis, Systemic Treatment, and Post-treatment Monitoring	N [2024], Y [2021]	The Taiwan Liver Cancer Association (TLCA) and the Gastroenterological Society of Taiwan, Taiwan	Evidence from BMJ 1999/Level 1,1a,1b,2,3,4; Evidence from BMJ 1999/1,1a,1b,2,3,4	Recommendation from BMJ 1999/Strong, moderate, considerable; Recommendation from BMJ 1999/A,B,C,D

AASLD, American Association for the Study of Liver Diseases; AGA, the American Gastroenterological Association; APASL, The Asian Pacific Association for the Study of the Liver; APPLE, the Asia-Pacific Primary Liver Cancer Expert; ASCO, American Society of Clinical Oncology; BCLC, Barcelona Clinic Liver Cancer; BMJ, British medical journal; EASL, European Association for the Study of the Liver; ESMO, European Society for Medical Oncology; EORTC, European Organization for Research and Treatment of Cancer; GRADE, the Grading of Recommendations, Assessment, Development and Evaluations; HCC, hepatocellular carcinoma; JSH, the Japan Society of Hepatology; KLCA, the Korean Liver Cancer Association; KPGRC: NCCN, NCC Korea Practice Guideline Revision Committee; National Comprehensive Cancer Network; TCLA, the Taiwan Liver Cancer Association.

### Overview of AGREE-II and evidence behind the guidelines and consensuses

Table 2 presents the domain scores of 23 clinical practice guidelines, considered 14 comprehensive guidelines, as evaluated using the AGREE-II instrument. Seven of these guidelines were revised to present results for the latest version. The scope and purpose domain pertains to the clarity and comprehensiveness of the guidelines’ scope and objectives. The scores ranged from 67.78 to 100%, with the ASCO guideline in 2021, EASL guideline in 2018, and Korea guideline in 2022 achieving the highest scores. The stakeholder involvement domain evaluates the extent of relevant stakeholders’ participation in the guideline development process. The scores ranged from 51.11 to 94.44%, with the ASCO guideline in 2021 achieving the highest score. The rigor of development domain assesses the methodological rigor employed in gathering and synthesizing evidence to formulate recommendations, with scores ranging from 63.33 to 97.92%. The clarity of presentation domain pertains to the clarity and accessibility of the guidelines in presentation, with scores ranging from 84.44% to 100. The applicability domain evaluates the extent to which the guidelines consider practical implementation and application, with scores varying from 73.33% to 100. The editorial independence domain pertains to the extent to which the guidelines remain free from undue influence, with scores ranging from 80% to 100. Overall, the Japan guideline in 2023 achieved the highest overall score of 6.80. The scores from each assessor are listed in Table S1 (Supplemental Digital Content 4, http://links.lww.com/JS9/D134).

**Table 2:** Domain scores (%) of the 16 clinical practice guidelines assessed using the AGREE-II instrument.

Domain	AASLD (2023)	AGA (2022)	APASAL (2017)	ASCO (2024)	Canada (2021)	Chinese (2023)	ESAL (2018)	ESMO (2021)	Japan (2023)	Korea (2022)	NCCN (2024)	Taiwan (2024)	APPLE (2020)	BCLC (2022)
Domain 1: scope and purpose	91.67%	92.22%	83.33%	**100.00%**	98.89%	88.89%/	**100.00%**	67.78%	94.44%	**100.00%**	81.11%	94.44%	95.56%	72.22%
Domain 2: stakeholder involvement	69.44%	83.33%	61.11%	**94.44%**	71.11%	58.33%/	78.89%	51.11%	80.56%	76.67%	61.11%	71.30%	63.33%	62.22%
Domain 3: rigor of development	67.71%	88.75%	76.25%	96.25%	70.42%	57.99%	88.75%	68.75%	**97.92%**	93.75%	88.54%	**97.92%**	65.42%	67.08%
Domain 4: clarity of presentation	100.00%	95.56%	95.56%	97.78%	84.44%	80.56%	95.56%	96.67%	100.00%/	100.00%	97.22%	97.22%	91.11%	**100.00%**
Domain 5: applicability	83.33%	73.33%	78.33%	80.0%	78.33%	77.08%	**86.67%**	85.00%	**100.00%**	80.00%	77.50%	79.86%	73.33%	74.17%
Domain 6: editorial independence	100.00%	91.67%	83.33%	91.67%	91.67%	100.00%	**93.33%**	80.00%	100.00%	100.00%	100.00%	100.00%	86.67%	88.33%
Overall (range 1~7)	6.09	6.21	5.72	6.78	5.76	5.33	6.39	5.43	**6.80**	6.37	5.80	6.41	5.54	5.47

AASLD, American Association for the Study of Liver Diseases; AGA, the American Gastroenterological Association; APASL, The Asian Pacific Association for the Study of the Liver; APPLE, the Asia-Pacific Primary Liver Cancer Expert; ASCO, American Society of Clinical Oncology; BCLC, Barcelona Clinic Liver Cancer; EASL, European Association for the Study of the Liver; ESMO, European Society for Medical Oncology; NCCN, National Comprehensive Cancer Network.

We included guidelines that provide explicit recommendations for the treatment of early-stage HCC, focusing particularly on RFA and surgical resection. It is important to note that while the ASCO guideline was included due to its comprehensive coverage and high AGREE-II score, it does not offer direct comparisons between RFA and surgical resection. This inclusion was made to encompass a broad perspective on the current landscape of HCC treatment guidelines, acknowledging that some highly regarded guidelines like ASCO prioritize a more generalized approach over specific treatment comparisons.

### Comparisons between recommendations

Table 3 summarizes the recommendations from 23 comprehensive clinical practice guidelines, focusing specifically on the decision-making between RFA and surgical resection for managing early-stage HCC. These guidelines exhibit variability, with some favoring RFA, others advocating surgical resection, and some maintaining a neutral stance. To enhance our visualizations, we employ PlantUML—a tool that generates Unified Modeling Language (UML) diagrams from textual inputs, as detailed in Table S3 (Supplemental Digital Content 5, http://links.lww.com/JS9/D135). Additionally, Table S2 (Supplemental Digital Content 6, http://links.lww.com/JS9/D136) presents a comparative analysis of updated guidelines from major liver societies, such as the American Association for the Study of Liver Diseases (AASLD), Chinese guidelines, Japanese guidelines, and others. This table highlights notable shifts in guideline recommendations over recent years, emphasizing an increasing recognition of the efficacy of RFA, particularly for patients with small, localized tumors who are ineligible for or decline surgical options. The table also illustrates the continued strong recommendation for surgical resection, especially in patients with resectable early-stage HCC and well-compensated cirrhosis, reflecting a high level of evidence supporting this modality.

**Table 3:** Recommendations from 23 clinical practice guidelines.

Category	Recommendations
RFA_A	Child’s A or B cirrhosis, HCC ≤2 cm 1
RFA_B	BCLC 0 2
RFA_C	CNLC 1a, 1b 3
RFA_D	HCC ≤2 cm 4
RFA_E	Single nodule<2 cm 5
RFA_F	HCC<2 cm 6
RFA_G	BCLC A, unresectable HCC 7
RFA_H	Solitary tumor ≤5 cm or early-stage HCC ≤3 cm, ineligible or decline surgery 8
Similar_A	Child’s A or B, HCC ≤3 cm 9
Similar_B	BCLC 0 10
Similar_C	Single nodular HCC ≤3 cm 11
Similar_D	BCLC 0, HCC<3 cm 12
Similar_E	Child’s A or B without metastasis and vascular invasion, three HCCs ≤3 cm 13
Similar_F	Solitary HCC or HCC<3 cm 14
Similar_G	BCLC 0, single tumor<2 cm 15
Similar_H	Within the Milan criteria 16
Similar _I	No special description of conditions 17
Similar_J	HCC ≤3 cm 18
Surgery_A	Child’s A, resectable T1 or T2 19
Surgery_B	BCLC A 20
Surgery_C	Child’s A, BCLC (0 or A), HCC<2 cm, ECOG 0 21
Surgery_D	CNLC 1a or 1b, Child’s A or B, HCC<5 cm 22
Surgery_E	Solitary HCC 23
Surgery_F	Recurrent HCC 24
Surgery_G	Localized HCC without cirrhosis or well-compensated with cirrhosis 25
Surgery_H	Generally 26

Footnotes:

1 Source: APASL 2017

2 Source: BCLC 2022, ESMO 2021, Taiwan 2021

3 Source: Chinese 2020, Chinese 2023

4 Source: Chinese 2020, Chinese 2023

5 Source: EASL 2018

6 Source: ESMO 2021

7 Source: Taiwan 2021

8 Source: AASLD 2023

9 Source: APASL 2017

10 Source: EASL 2018

11 Source: Korea 2019

12 Source: ESMO 2021

13 Source: Japan 2019, Japan 2023

14 Source: NCCN 2021

15 Source: Taiwan 2021

16 Source: Korea 2022

17 Source: NCCN 2024

18 Source: Chinese 2023

19 Source: AASLD 2018

20 Source: BCLC 2022

21 Source: Canada 2021

22 Source: Chinese 2020

23 Source: Japan 2019

24 Source: Chinese 2023, NCCN 2021

25 Source: AASLD 2023

26 Source: Chinese 2023

BCLC, Barcelona Clinic Liver Cancer; CNLC, Chinese Liver Cancer; ECOG, Eastern Cooperative Oncology Group; HCC, hepatocellular carcinoma; RFA, radiofrequency ablation.

Guidelines favoring RFA for early HCC treatment typically specify criteria such as patients with Child-Pugh A or B cirrhosis and HCC tumors measuring ≤2 cm, classified as BCLC 0, or stages CNLC 1a or 1b. RFA is also advised for patients with a solitary tumor ≤5 cm or early-stage HCC ≤3 cm who are ineligible for or decline surgery, as well as for those with HCC tumors ≤2 cm, a single nodule <2 cm in size, or unresectable HCC categorized as BCLC A.

Guidelines that adopt a neutral stance generally apply to patients with Child-Pugh A or B cirrhosis and HCC tumors measuring ≤3 cm. A neutral approach is also suggested for patients with HCC ≤3 cm, HCC <2 or 3 cm classified as BCLC 0, and those with Child-Pugh A or B cirrhosis without metastasis and vascular invasion, and those with up to three HCCs measuring ≤3 cm in size. Additionally, a neutral position is held for patients with solitary HCC or HCC tumors <3 cm in size or a single tumor measuring ≤3 cm. Some guidelines also recommend a neutral stance for patients meeting the Milan criteria (a single tumor ≤5 cm or less than three tumors ≤3 cm).

Guidelines recommending surgical resection for early HCC treatment include those advising surgery for patients with Child-Pugh A cirrhosis and resectable T1 or T2 tumors, stage BCLC A, or having Child-Pugh A cirrhosis with stages BCLC 0 or A, HCC tumors <2 cm in size, and an Eastern Cooperative Oncology Group (ECOG) performance status of 0. Surgical resection is also suggested for patients with CNLC 1a or 1b staging with Child-Pugh A or B cirrhosis, and HCC tumors <5 cm. Additional endorsements for surgical resection include patients with solitary HCC and those with recurrent HCC, and localized HCC without cirrhosis or with well-compensated cirrhosis. One guideline generally advocates surgical resection as a priority.

**Figure 2:**
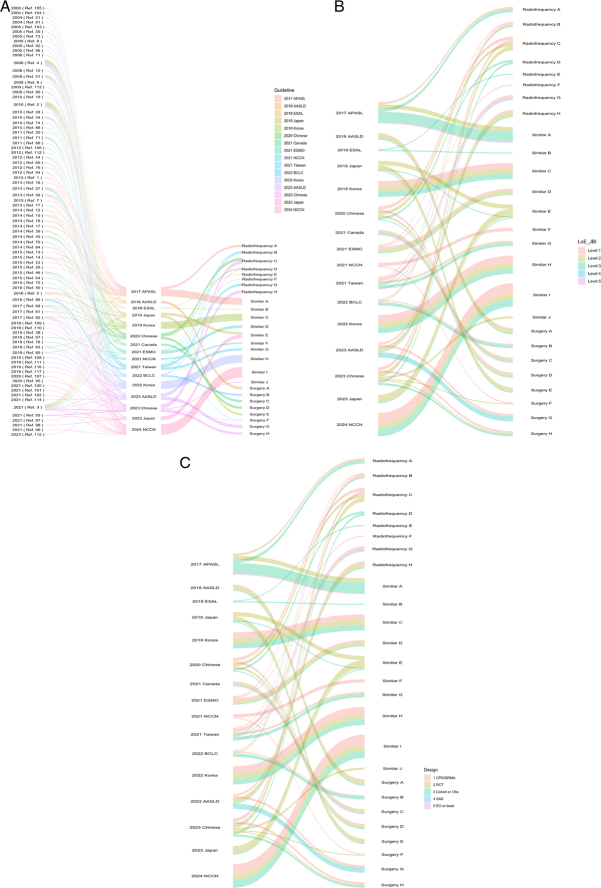
Clinical guideline recommendations in relation to evidence levels. (A) Relationship between references, guidelines, and recommendations; (B) levels of evidence between guidelines and recommendations; (C) research design between guidelines and recommendations.

Figure 2 presents a comprehensive visualization of the evidence strength and quality supporting the recommendations related to the management of early-stage HCC. The figure offers an overview of the evidence levels associated with each recommendation identified in Table 3. Figure 2A depicts a comprehensive analysis of 117 references from 16 distinct guidelines, which were used to develop recommendations for the choice between RFA and surgical resection for early-stage HCC management. Figure 2B graphically represents the evidence levels corresponding to the guidelines and their respective recommendations, evaluated using JBI Level of Evidence, wherein red indicates level 1 evidence and purple indicates level 5 evidence. Furthermore, Figure 2C presents a detailed breakdown of the types of studies cited in each reference that contributed to the formulation of the recommendations. These encompass systematic reviews and meta-analyses, RCTs, cohort studies, single-arm studies, expert opinions, and books.

Further investigation showed some significant findings among levels of evidence in each recommendation category. The category regarding the recommendation of surgery for HCC primarily relied on level 1 evidence (*χ* ^2^ test=27.429; *P*<0.001), with few sources having evidence at level 2 or level 4 (*χ* ^2^ test=6.857; *P*=0.035). The category regarding the recommendation of radiofrequency procedures for HCC primarily relied on level 1 (*χ* ^2^ test=9.720; *P*=0.007) and level 3 evidence (*χ* ^2^ test=7.053; *P*=0.032), with few sources having evidence at level 2 or level 4 (*χ* ^2^ test=8.333; *P*=0.016). The category of recommendations for both surgery and radiofrequency procedures primarily drew on evidence at level 1 (*χ* ^2^ test=19.44; *P*<0.001) and level 3 (*χ* ^2^ test=11.76; *P*<0.001), and evidence at level 2 (*χ* ^2^ test=16.67; *P*<0.001), and level 4 (*χ* ^2^ test=14.11; *P*=0.002) was conspicuously absent.

## Discussion

### Summary of findings

Our analysis illuminated significant shifts in the clinical guidelines for the management of early-stage HCC. Over the years, there has been a notable increase in the endorsement of RFA for patients with specific tumor characteristics, mirroring broader acceptance of its efficacy and safety in less invasive treatment scenarios. Conversely, surgical resection continues to be highly recommended, particularly for patients with resectable tumors and good liver function, reflecting its established curative potential. These findings from our systematic review of updated guidelines suggest a trend towards more personalized treatment strategies, allowing for greater flexibility in choosing between RFA and surgery based on individual patient conditions and the latest clinical evidence.

While our review meticulously evaluates existing guidelines and the underlying evidence for the treatment of early-stage HCC with a focus on RFA and surgical resection, it is important to clarify our position on the current evidence base. We recognize the substantial contributions of existing high-quality studies, including RCTs, to our understanding of these treatments’ effectiveness. These studies provide critical insights that inform current clinical practice and guideline recommendations. However, our analysis also identifies notable inconsistencies across guidelines, not as a critique of the existing evidence per se, but as an observation highlighting areas where further clarity could benefit clinical decision-making. In evaluating the clinical guidelines for early-stage HCC treatment, our study also considers the impact of different healthcare systems and medical infrastructures on the observed discrepancies in these guidelines. The variability in treatment accessibility and outcomes across regions suggests that guidelines are often tailored to reflect local healthcare capacities and needs. Therefore, it is important to recognize that these guidelines do not necessarily need to converge on a uniform statement but should instead be contextually appropriate, providing the best possible care under varying conditions.

### Clinical considerations/implications

RFA offers advantages over surgical resection, including lower complication rates and minimal damage to surrounding healthy liver tissue, thereby preserving more viable liver tissue^43^. In appropriately selected patients, RFA yields comparable survival rates to surgical resection^44^. For selected patients, RFA can achieve survival rates comparable to surgical resection, with complete response rates between 70 and 90% significantly linked to improved overall survival (median overall survival of 60 months)^3^. However, the 5-year recurrence rate after RFA can reach 50–70%, with incomplete ablation particularly problematic for irregular lesions or in the presence of intrahepatic metastasis, multicenter recurrence, and small satellite nodules^45^. Incomplete tumor ablation can occur with RFA, particularly when dealing with irregular lesions or conditions such as intrahepatic metastasis, multicenter recurrence, and small satellite nodules. Various factors contribute to this, including off-center positioning of the RFA electrode due to restricted access windows, dissipation of RFA heat near large blood vessels, and the need to safeguard adjacent vital organs, leading to an increased risk of rapid tumor progression in such cases^46^.

Recent advancements in surgical methodologies have precipitated an increasing inclination toward minimally invasive approaches for the resection of small HCC lesions. Minimally invasive surgery (MIS) for HCC not only facilitates the excision of microscopic metastases and sites of microvascular invasion potentially overlooked during preoperative or intraoperative assessments but also serves as a critical mechanism for mitigating recurrence risks^47^. Studies have highlighted the benefits of such approaches, including reduced postoperative pain, shorter hospital stays, and quicker recovery times, without compromising the oncological outcomes compared to traditional open surgery^48^. The decision-making matrix for the selection of an optimal treatment modality for HCC is complex, necessitating the consideration of an array of variables, including, but not limited to, tumor dimensions and locational attributes, patient health status, and liver functional capacity. The implementation of surgical resection, particularly within cirrhotic patient populations, introduces considerable challenges due to the amplified procedural stress and potential trauma to already compromised hepatic tissues, coupled with a reduced capacity for liver regeneration^49^. A systematic review and meta-analysis posit that surgical resection is optimally reserved for patients exhibiting robust performance statuses, adequate hepatic reserves, and anatomically resectable lesions, whereas RFA may present a more suitable intervention for alternative patient cohorts^43^. The preference for MIS in the treatment of small HCC further complicates the decision-making process between RFA and surgical resection. While RFA remains a valuable option for patients with small tumors and those who may not be ideal candidates for surgery, the advancements in MIS techniques suggest that surgical resection could become more accessible and appealing for a broader patient population. Therefore, the choice between RFA and surgical resection must consider not only the tumor characteristics and patient’s liver function but also the available surgical expertise and technological infrastructure.

Furthermore, the discrepancies observed in the guidelines for early-stage HCC treatment, especially between RFA and surgical resection, can often be attributed to variations in healthcare systems and medical infrastructures that are influenced by regional and racial differences. For example, regions with advanced healthcare infrastructure might show a preference for surgical interventions due to better access to required technologies and skilled specialists, whereas regions with limited resources might favor less resource-intensive treatments like RFA^43^. Additionally, the adoption of new technologies such as navigation-assisted ablation comes with a learning curve that might influence treatment choices differently across regions^44^. This diversity in healthcare contexts highlights the challenges of adopting a uniform approach in guideline development and stresses the importance of guidelines being adaptable to reflect not only the best scientific evidence but also the specific healthcare realities of different regions, taking into account racial and ethnic factors that might affect disease presentation and treatment outcomes. These considerations are vital for developing guidelines that are both scientifically sound and pragmatically applicable across various global contexts.

It is important to clarify that this study does not suggest a preferential recommendation for either treatment modality. For oncologists and patients making treatment decisions, direct comparisons of patient outcomes from high-quality RCTs, systematic reviews, and meta-analyses would offer more definitive guidance. Consequently, our findings highlight the necessity for more comprehensive and directly comparable evidence to inform future guidelines, ensuring that recommendations are both clear and grounded in robust evidence.

### Reflections on the process from evidence to recommendation

The decision-making process between RFA and liver resection involves multiple factors, including tumor size, BCLC staging, Child-Pugh score, and ECOG performance status. Guidelines integrate these factors, offering a comprehensive approach to treatment selection. Notably, RFA recommendations often stem from studies with lower evidence strength, highlighting the challenges of conducting RCTs to fully assess RFA’s efficacy and outcomes. Despite this, observational studies provide compelling evidence supporting RFA recommendations. Continued research is expected to bolster the evidence base for both treatments, improving future guidelines’ precision and relevance. However, the lack of stakeholder input in the guidelines underscores the importance of shared decision-making when both RFA and surgical resection are considered viable options. Future guideline development efforts should aim to provide flexible recommendations that account for variations in medical infrastructure and healthcare systems.

### Limitations

While our review provides comprehensive insights from European and Asia-Pacific guidelines, we acknowledge the limitation of not including guidelines from all geographic regions. Future systematic reviews could benefit from an expanded scope that includes guidelines from the Americas, Africa, and other areas to ensure a more globally comprehensive perspective on the management of HCC.

While we emphasize the need for large-scale RCTs to robustly evaluate the effectiveness of RFA in treating early-stage HCC, it is important to recognize the barriers that currently limit such studies. These include logistical challenges, high costs, ethical considerations given existing treatment alternatives, and the difficulty in enrolling patients who meet specific criteria. Additionally, we acknowledge the existence of high-quality studies, including existing RCTs, that have already provided valuable insights into comparing RFA and surgical resection. These studies illustrate the ongoing efforts and advancements in understanding the best treatment modalities for HCC, contributing to a nuanced and balanced view of the current evidence base.

## Conclusion

Our systematic review of clinical guidelines for the treatment of early-stage HCC has revealed significant inconsistencies in the recommendations for RFA and surgical resection. We identified 26 distinct recommendations, finding that while surgical resection often receives backing from high-level evidence such as RCTs, RFA is increasingly recognized for its effectiveness in specific patient scenarios, particularly those with small, localized tumors where surgery poses significant risks. The variation in evidence, high-quality comparative studies to clarify and harmonize treatment strategies. Future guideline development must not only uphold methodological rigor but also broaden stakeholder engagement to include diverse perspectives, particularly from patient groups. Additionally, guidelines should be dynamically adapted to reflect regional differences and the varying accessibility of medical resources, ensuring that they support clinicians in making well-informed treatment decisions tailored to the needs of patients with early-stage HCC. This approach will facilitate the development of comprehensive, patient-centered guidelines that effectively address the current challenges in managing this complex disease.

## Ethical approval

Not applicable.

## Consent

Not applicable.

## Source of funding

This study was funded by the Taiwan Ministry of Health and Welfare (Grant Number: MOHW113-TDU-B-221-134007) and VGH, TSGH, AS Joint Research Program (Grant Number: VTA113-V1-1-1).

## Author contribution

T.W.H. and Y.N.K.: designed and supervised the study; T.Y.L., Y.C.H., C.C.Y., C.C., C.Y.W., L.Y.L., T.W.H., and Y.N.K.: searched for and selected the trials; T.W.H. and Y.N.K.: did the statistical analyses and wrote the draft. All authors contributed to interpreting the results during the investigator meeting and revising the manuscript. The corresponding author and the first author had full access to all the data in the study and had final responsibility for the decision to submit for publication. C.Y.W., T.W.H., and Y.N.K.: have accessed and verified the data.

## Conflicts of interest disclosure

The authors declare no conflict of interest.

## Research registration unique identifying number (UIN)

Chun-Ying Wu, Yi-No Kang, and Tsai-Wei Huang, serving as both the first authors and corresponding authors, take full responsibility for the entirety of the work and the conduct of the study. They had complete access to the data and maintained control over the decision to publish.

## Guarantor

PROSPERO (CRD42022342266).

## Data availability statement

Research data are stored in an institutional repository and will be shared with the corresponding author upon request. Patients or the public WERE NOT involved in our research’s design, conduct, reporting, or dissemination plans.

## Provenance and peer review

Not commissioned, externally peer-reviewed.

## Supplementary Material
