# Supplementary material for: Clinical guidelines for early hepatocellular carcinoma treatment options: a systematic review and bibliometric analysis
Source: Int J Surg. 2024 Jul 23;110(11):7234–44. doi: 10.1097/JS9.0000000000001950 (PMC11573054; doi:10.1097/JS9.0000000000001950)
Supplement: Supplementary file 2 [file js9-110-7234-s002.docx]

**Identification of studies via other methods**

**Identification of studies via databases and registers**

Records identified from:

Organisations (n = 6)

Records removed *before screening*:

Duplicate records removed

(n = 9332)

Not guideline or consensus

(n = 26870)

Records identified from:

PubMed (n = 9374)

Embase (n = 27960)

Cochrane Library (n = 1472)

**Identification**

Records screened

(n = 2604)

Records excluded by screening title and abstract

(n = 2099)

Reports not retrieved

(n = 0)

Reports sought for retrieval

(n = 6)

Reports sought for retrieval

(n = 505)

Reports not retrieved

(n = 0)

**Screening**

Reports excluded:

Not complying PICO (n = 280)

Before 2017 (n = 185)

Not in English (n = 3)

Region out of interest (n = 12)

Old version of guidelines (n = 7)

Reports assessed for eligibility

(n = 6)

Reports excluded

(n = 0)

Reports assessed for eligibility

(n = 505)

Guidelines included in review

(n = 14)

Reports of included guidelines

(n = 23)

**Included**

Figure 1. Study selection process.
