# Supplementary material for: Clinical guidelines for early hepatocellular carcinoma treatment options: a systematic review and bibliometric analysis
Source: Int J Surg. 2024 Jul 23;110(11):7234–44. doi: 10.1097/JS9.0000000000001950 (PMC11573054; doi:10.1097/JS9.0000000000001950)
Supplement: Supplementary file 4 [file js9-110-7234-s004.docx]

Table S1. Quality assessment of HCC clinical practice guidelines for the six domains of the AGREE-II Instrument (D1 to D6) and the overall impression of the five assessors

| Domain | AASLD  (2023) | AGA  (2022) | APASAL  (2017) | ASCO  (2024) | Canada  (2021) | Chinese  (2023) | ESAL  (2018) | ESMO  (2021, 2018) | Japan  (2023) | Korea  (2022) | NCCN  (2024) | Taiwan  (2024) | APPLE  (2020) | BCLC  (2022) |
| --- | --- | --- | --- | --- | --- | --- | --- | --- | --- | --- | --- | --- | --- | --- |
| D1 |  |  |  |  |  |  |  |  |  |  |  |  |  |  |
| 1 | 7.00 | 6.80 | 6.40 | 7.00 | 6.80 | 7.00 | 7.00 | 5.40 | 7.00 | 7.00 | 5.00 | 7.00 | 6.80 | 5.60 |
| 2 | 7.00 | 6.80 | 6.40 | 7.00 | 7.00 | 7.00 | 7.00 | 5.40 | 7.00 | 7.00 | 5.00 | 6.50 | 6.80 | 5.20 |
| 3 | 5.50 | 6.00 | 5.20 | 7.00 | 7.00 | 5.00 | 7.00 | 4.40 | 6.00 | 7.00 | 6.00 | 7.00 | 6.60 | 5.20 |
| D2 |  |  |  |  |  |  |  |  |  |  |  |  |  |  |
| 4 | 5.00 | 6.20 | 6.20 | 6.00 | 6.60 | 5.00 | 6.60 | 5.40 | 5.50 | 5.50 | 5.00 | 6.00 | 5.60 | 5.20 |
| 5 | 5.00 | 5.00 | 2.60 | 7.00 | 3.80 | 4.00 | 3.60 | 2.80 | 6.00 | 2.00 | 2.00 | 3.50 | 3.80 | 3.80 |
| 6 | 5.50 | 6.80 | 5.20 | 7.00 | 5.40 | 4.50 | 7.00 | 4.00 | 6.00 | 7.00 | 7.00 | 6.00 | 5.00 | 5.20 |
| D3 |  |  |  |  |  |  |  |  |  |  |  |  |  |  |
| 7 | 6.00 | 5.80 | 4.20 | 7.00 | 6.40 | 2.50 | 4.60 | 3.80 | 7.00 | 7.00 | 6.00 | 7.00 | 3.60 | 4.20 |
| 8 | 3.00 | 6.20 | 5.40 | 7.00 | 4.00 | 2.50 | 6.80 | 4.60 | 7.00 | 6.50 | 6.00 | 7.00 | 3.40 | 3.60 |
| 9 | 6.00 | 6.80 | 6.40 | 7.00 | 4.60 | 3.00 | 6.80 | 6.20 | 7.00 | 6.50 | 6.50 | 7.00 | 5.00 | 5.20 |
| 10 | 6.00 | 6.20 | 6.20 | 7.00 | 4.20 | 3.00 | 6.60 | 3.80 | 7.00 | 6.50 | 6.50 | 7.00 | 5.80 | 3.80 |
| 11 | 6.00 | 6.60 | 6.20 | 7.00 | 5.80 | 4.00 | 6.00 | 6.20 | 7.00 | 6.50 | 6.50 | 6.00 | 6.20 | 6.60 |
| 12 | 6.00 | 6.80 | 6.40 | 7.00 | 6.00 | 5.50 | 6.80 | 6.20 | 7.00 | 6.50 | 6.50 | 7.00 | 6.00 | 6.40 |
| 13 | 5.00 | 5.60 | 5.20 | 7.00 | 6.00 | 5.00 | 6.20 | 4.40 | 6.00 | 5.50 | 6.00 | 7.00 | 5.60 | 5.60 |
| 14 | 7.00 | 6.60 | 4.60 | 7.00 | 4.80 | 7.00 | 6.80 | 5.80 | 7.00 | 7.00 | 6.50 | 7.00 | 3.80 | 4.80 |
| D4 |  |  |  |  |  |  |  |  |  |  |  |  |  |  |
| 15 | 7.00 | 6.80 | 6.80 | 7.00 | 6.00 | 7.00 | 6.80 | 6.80 | 7.00 | 7.00 | 7.00 | 7.00 | 6.60 | 7.00 |
| 16 | 7.00 | 6.60 | 6.80 | 7.00 | 6.20 | 7.00 | 6.60 | 6.60 | 7.00 | 7.00 | 7.00 | 7.00 | 6.60 | 7.00 |
| 17 | 7.00 | 6.80 | 6.60 | 7.00 | 6.00 | 7.00 | 6.80 | 7.00 | 7.00 | 7.00 | 6.50 | 6.50 | 6.20 | 7.00 |
| D5 |  |  |  |  |  |  |  |  |  |  |  |  |  |  |
| 18 | 6.00 | 4.60 | 4.60 | 6.00 | 5.40 | 4.50 | 6.00 | 6.00 | 7.00 | 6.00 | 4.00 | 5.00 | 4.80 | 4.40 |
| 19 | 6.00 | 6.00 | 6.20 | 6.00 | 6.00 | 6.00 | 6.40 | 6.20 | 7.00 | 6.00 | 5.00 | 6.00 | 6.20 | 6.60 |
| 20 | 6.00 | 5.00 | 6.20 | 6.00 | 5.40 | 6.00 | 6.40 | 6.00 | 7.00 | 4.50 | 4.50 | 5.00 | 4.80 | 4.80 |
| 21 | 6.00 | 6.00 | 5.80 | 6.00 | 6.00 | 6.00 | 6.00 | 6.20 | 7.00 | 6.00 | 5.00 | 6.00 | 5.80 | 6.00 |
| D6 |  |  |  |  |  |  |  |  |  |  |  |  |  |  |
| 22 | 7.00 | 6.20 | 5.20 | 7.00 | 6.20 | 7.00 | 6.40 | 5.20 | 7.00 | 7.00 | 7.00 | 7.00 | 5.80 | 5.80 |
| 23 | 7.00 | 6.80 | 6.80 | 7.00 | 6.80 | 7.00 | 6.80 | 6.40 | 7.00 | 7.00 | 7.00 | 7.00 | 6.60 | 6.80 |
| Overall | 6.09 | 6.21 | 5.72 | 6.78 | 5.76 | 5.33 | 6.39 | 5.43 | 6.80 | 6.37 | 5.80 | 6.41 | 5.54 | 5.47 |

Domains are as follows: D1, scope and purpose; D2, stakeholder involvement; D3, rigor of involvement; D4, clarity of presentation; D5, applicability; and D6, editorial independence. All the 23 items of the Appraisal of Guidelines Research and Evaluation II (AGREE-II) instrument are rated on a 7-point scale from 7 (*strongly agree*) down to 1 (*strongly disagree*). Overall: the numbers in the overall row represent the average of the scores assigned by four assessors.

AASLD: American Association for the Study of Liver Diseases; AGA: the American Gastroenterological Association; APASL: The Asian Pacific Association for the Study of the Liver; APPLE: the Asia-Pacific Primary Liver Cancer Expert; ASCO: American Society of Clinical Oncology; BCLC: Barcelona Clinic Liver Cancer; EASL: European Association for the Study of the Liver; ESMO: European Society for Medical Oncology; NCCN: National Comprehensive Cancer Network
