# Supplementary figures and images for: Clinical guidelines for early hepatocellular carcinoma treatment options: a systematic review and bibliometric analysis
Source: Int J Surg. 2024 Jul 23;110(11):7234–44. doi: 10.1097/JS9.0000000000001950 (PMC11573054; doi:10.1097/JS9.0000000000001950)

Table S3. Visualized Recommendations


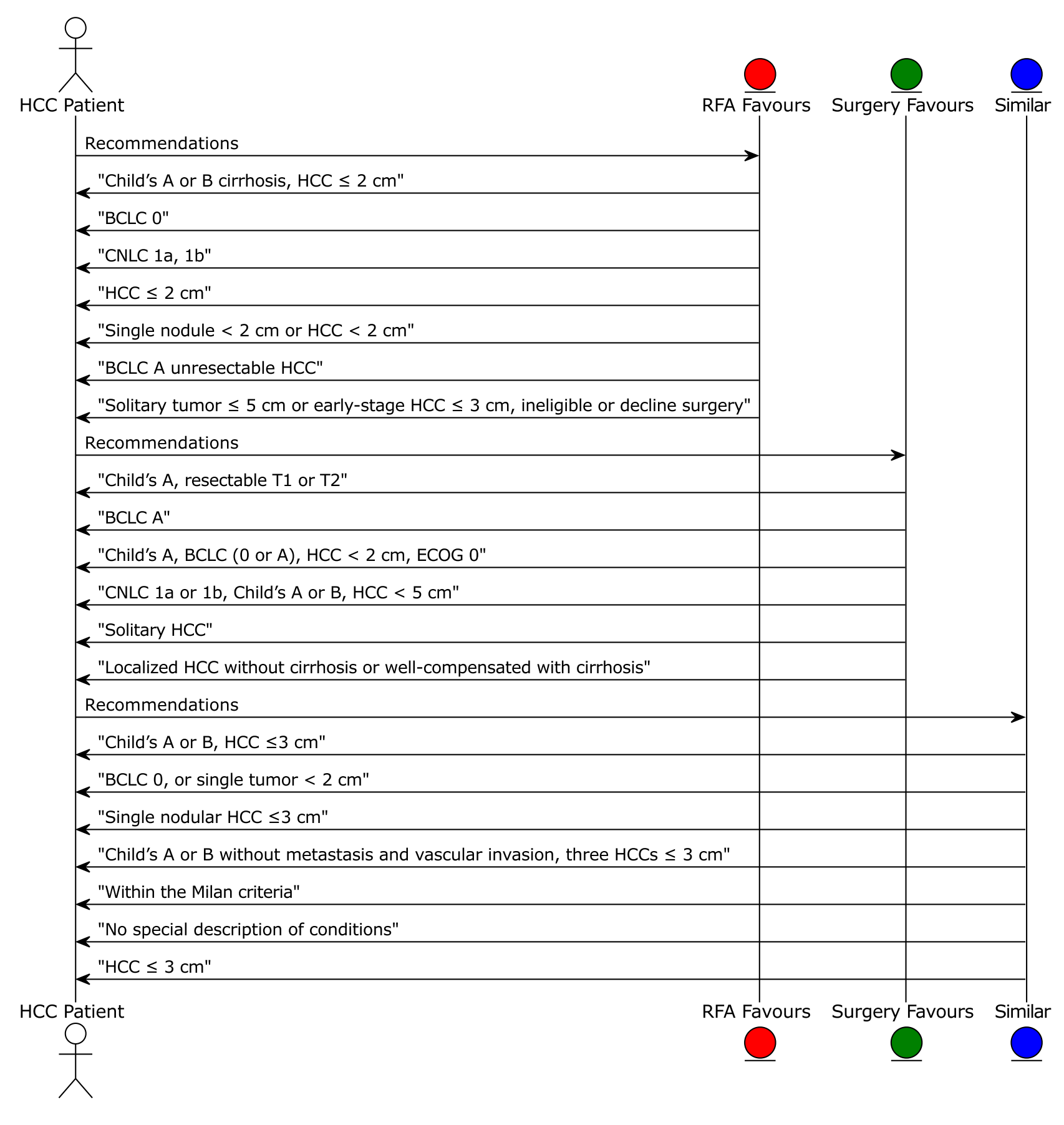

Supplement: Supplementary file 5 [file js9-110-7234-s005.docx]
