# Supplementary material for: Clinical guidelines for early hepatocellular carcinoma treatment options: a systematic review and bibliometric analysis
Source: Int J Surg. 2024 Jul 23;110(11):7234–44. doi: 10.1097/JS9.0000000000001950 (PMC11573054; doi:10.1097/JS9.0000000000001950)
Supplement: Supplementary file 6 [file js9-110-7234-s006.docx]

Table S2. Comparative Analysis of Updated Clinical Guidelines: Focusing on Radiofrequency Ablation (RFA) and Surgical Treatments for Hepatocellular Carcinoma

| **AASLD Guidelines Update: 2018 vs. 2023** | |
| --- | --- |
| AASLD 2018 | AASLD 2023 |
| The AASLD suggests that adults with Child's A cirrhosis and resectable T1 or T2 HCC undergo resection over radiofrequency ablation (RFA). | - Surgical resection should be the treatment of choice for localized HCC in the absence of underlying cirrhosis (Level 2, Strong Recommendation). - In patients with cirrhosis, surgical resection should be considered the treatment of choice for patients with limited tumor burden, well-compensated cirrhosis without clinically significant portal hypertension, and an adequate FLR (Level 2, Strong Recommendation). - Minimally invasive liver resection (laparoscopic and robotic) may be performed to enhance recovery and lower risk of perioperative morbidity in selected patients (Level 3, Weak Recommendation). - Patients with solitary tumors ≤5 cm should be treated with curative intent using local ablative therapies if **ineligible for or they decline surgical therapy** (Level 1, Strong Recommendation). - Thermal ablation (radiofrequency or microwave ablation) should be considered the treatment of choice for patients with early-stage HCC ≤3 cm who are **ineligible for or decline surgery** (Level 1, Strong Recommendation). |
| **Chinese Guidelines Update: 2019 vs. 2023** | |
| Chinese 2019 | Chinese 2023 |
| 1. Local ablation therapy is suitable for patients with CNLC Stage Ia and a proportion of patients with Stage Ib HCC (i.e., solitary tumors with a diameter of ≤5 cm or 2–3 tumors with maximum diameter ≤3 cm); no invasion of blood vessels and bile ducts; without adjacent organ invasion or distal metastasis and Child-Pugh Grade A/B liver function. Outcomes of ablation therapy are comparable to those of radical resection in these selected patients. TACE combined with ablation can be used for inoperable solitary or multiple tumors with a diameter of 3–7 cm. Postoperative adjuvant therapy with sorafenib is not recommended in patients undergoing radical ablation therapy.  2. For patients with resectable early-stage HCC, RFA is associated with similar or slightly lower tumor-free survival and overall survival, but lower incidence of complications and shorter hospital stay than surgical resection. For solitary HCC with a diameter ≤2 cm, RFA has been shown to have similar or superior efficacy to surgical resection, in particular for central HCC. For patients with unresectable early-stage HCC, RFA can lead to curative outcomes and should be recommended as the first-line treatment. | - Ablation therapy is suitable for patients with CNLC stage Ia and some patients with stage Ib HCC (i.e., solitary tumors with a diameter of ≤5 cm or 2–3 tumors with maximum diameter ≤3 cm) to obtain a curative outcome. TACE combined with ablation may be used for inoperable solitary or multiple tumors with a diameter of 3–7 cm. - For tumors with a diameter ≤3 cm, the tumor-free and OS rates of ablation therapy are similar to, or slightly lower than, those of surgical resection, but the complication rate and length of hospital stay are lower compared with surgical resection. For a single HCC lesion ≤2 cm in diameter, the efficacy of ablation therapy is similar to that of surgical resection, especially for central HCC. - Statement: For tumors with a diameter ≤3 cm, the tumor-free and OS rates of ablation therapy are similar to, or slightly lower than, those of surgical resection, but the complication rate and length of hospital stay are lower compared with surgical resection. For a single HCC lesion ≤2 cm in diameter, the efficacy of ablation therapy is similar to that of surgical resection, especially for central HCC. - Explanation: For patients with resectable early-stage HCC, RFA is associated with similar or slightly lower tumor-free survival and OS than surgical resection, with a lower incidence of complications and shorter hospital stay (evidence level 1, recommendation A). For solitary HCC (particularly central solitary HCC) ≤2 cm in diameter, RFA has similar or superior efficacy to surgical resection (evidence level 3, recommendation A). - The recommended treatment for patients with CNLC stages Ia, Ib, and IIa HCC and enough LFR is surgical resection. Previous studies have shown no significant differences in the efficacy of surgical resection and radiofrequency ablation (RFA) for HCC ≤3 cm in diameter (evidence level 1, recommendation B). However, in recent studies, surgical resection was associated with a significantly lower local recurrence rate than RFA and better long-term outcomes (evidence level 1, recommendation A). Even for recurrent HCC, the prognosis following surgical resection remains better than that following RFA in selected patients (evidence level 2, recommendation B). |
| **Japan Guidelines Update: 2019 vs. 2023** | |
| Japan 2019 | Japan 2023 |
| Three treatments are recommended for HCC patients with Child-Pugh A/B liver function without extrahepatic metastasis or vascular invasion. First, either **surgical resection or radiofrequency ablation is recommended with no priority for up to three HCCs measuring ≤3 cm**; however, **surgical resection is recommended as first-line therapy for solitary HCC regardless of size**. Although there were four randomized controlled trials comparing surgery and radiofrequency ablation during the targeted period, their results were not reflected in this algorithm, because all of them had problems associated with study design or patient background. Based on the results of a nationwide large cohort study carried out by the Liver Cancer Study Group of Japan comparing the outcomes of hepatectomy, radiofrequency ablation, and percutaneous ethanol injection for solitary HCC ≤3 cm in size, which found the **better prognosis after hepatectomy, surgical resection is recommended as first-line therapy for solitary HCC.** | In the revised 5th JSH-HCC guidelines, the most significant changes in the treatment algorithm are that hepatectomy and radiofrequency ablation are now **equally recommended** for up to three HCCs ≤3 cm in size, and the ranking order of the recommended treatments for HCC with vascular invasion was created. As for the treatment of up to three small HCCs, randomized controlled trials (RCTs) conducted in Hong Kong and Japan (SURF trial) have been newly included, demonstrating that there was **no difference in prognosis after treatment between hepatectomy and radiofrequency ablation.** |
| **Korea Guidelines Update: 2019 vs. 2022** | |
| Korea 2019 | Korea 2022 |
| RFA has the **equivalent survival rate, a higher local tumor recurrence rate**, and a lower complication rate than hepatic resection in patients with a single nodular HCC ≤3 cm in diameter (A1) | In conclusion, for HCCs within the Milan criteria, hepatic resection has shown a lower recurrence rate than RFA and a higher postoperative complication rate; however, further studies are warranted to verify the difference in the survival rate. For single nodular HCCs <3 cm in diameter, RFA has an equivalent survival rate, higher LTP (local tumor progression) rate, and lower complication rate than hepatic resection. Therefore, it can be used as an alternative treatment for surgery if the location of HCC is ideal to perform RFA. |
| **NCCN Guidelines Update: 2021 vs. 2024** | |
| NCCN 2021 | NCCN 2024 |
| Resection is generally associated with better survival outcomes than RFA but is associated with more complications and morbidity from complications.  Subgroup analyses from one meta-analysis showed no significant differences in 1-year mortality and disease recurrence when including only studies with patients who had solitary or small tumors (.3cm).  One meta-analysis comparing RFA to resection in recurrent HCC (including 6 retrospective comparative studies) showed that 3- and 5-year DFS rates were greater for resection, relative to RFA. | - Studies that have compared RFA and resection have failed to provide conclusive evidence. RFA and liver resection in the treatment of patients with HCC have been compared in randomized prospective studies. The results of one randomized trial showed a significant survival benefit for resection over RFA in 235 patients with small HCC conforming to the Milan criteria. The 5-year OS rates were 54.8% and 75.6% for the RFA group and resection, respectively. The corresponding RFS rates for the two groups were 28.7% and 51.3%, respectively. However, more patients in the resection group were lost to follow-up than the RFA group. Conversely, other randomized studies demonstrated that percutaneous local ablative therapy with RFA is as effective as a resection for patients with early-stage disease (e.g., small tumors). These studies failed to show statistically significant differences in OS and DFS between the two treatment groups. In addition, in one of the studies, tumor location was an independent risk factor associated with survival. These studies, however, were limited by the small number of patients (180 patients and 168 patients, respectively) and the lack of a non-inferiority design. **Nevertheless, results from these studies support ablation as an alternative to resection in patients with small (<3 cm), properly located tumors.** - RFA has been compared to resection in some meta-analyses, which have shown that **resection is generally associated with better survival outcomes than RFA but is associated with more complications and morbidity from complications.** 6 retrospective comparative studies) showed that 3- and 5-year DFS rates were greater for resection, relative to RFA. - **Subgroup analyses from some retrospective studies suggest that tumor size is a critical factor in determining the effectiveness of RFA or resection.** Some investigators consider RFA as the first-line treatment in highly selected patients with HCC tumors that are less than or equal to 2 cm in diameter in an accessible location and away from major vascular and biliary structures and adjacent organs. |
